# Experience of extreme weather affects climate change mitigation and adaptation responses

**DOI:** 10.1007/s10584-016-1837-4

**Published:** 2016-10-24

**Authors:** Christina Demski, Stuart Capstick, Nick Pidgeon, Robert Gennaro Sposato, Alexa Spence

**Affiliations:** 1 Understanding Risk Research Group, School of Psychology, Cardiff University, Cardiff, CF10 3AT UK; 2 Horizon Digital Economy Research/School of Psychology, University of Nottingham, Nottingham, NG7 2TU UK

**Keywords:** Climate Change, Risk Perception, Behavioural Intention, Climate Risk, Epistemic Belief

## Abstract

The winter of 2013/2014 saw a series of severe storms hit the UK, leading to widespread flooding, a major emergency response and extensive media exposure. Previous research indicates that experiencing extreme weather events has the potential to heighten engagement with climate change, however the process by which this occurs remains largely unknown, and establishing a clear causal relationship from experience to perceptions is methodologically challenging. The UK winter flooding offered a natural experiment to examine this question in detail. We compare individuals personally affected by flooding (*n* = 162) to a nationally representative sample (*n* = 975). We show that direct experience of flooding leads to an overall increased salience of climate change, pronounced emotional responses and greater perceived personal vulnerability and risk perceptions. We also present the first evidence that direct flooding experience can give rise to behavioural intentions beyond individual sustainability actions, including support for mitigation policies, and personal climate adaptation in matters unrelated to the direct experience.

**Electronic supplementary material:**

The online version of this article (doi:10.1007/s10584-016-1837-4) contains supplementary material, which is available to authorized users.

## Introduction

This paper examines the effect of experiencing extreme weather on personal engagement with climate change. We address this by focusing on a specific series of flooding events that occurred in the UK in late 2013/early 2014. These events were brought about by a series of major storms hitting the UK in quick succession, leading to extensive and persistent flooding events across England and Wales. The impacts were consistent with projections for increased UK flood risk under climate change (Huntingford et al. 2014), led to a major emergency response, and prompted Prime Minister David Cameron and national media to attribute the events, at least in part, to climate change (BBC Online 2014). Subsequent climate model simulations indicate that the risks of extreme precipitation in Southern England at the time were heightened significantly by anthropogenic warming (Schaller et al. 2016).

The notion that a person’s ‘experience’ with weather-related phenomena provides a potentially important route to engagement with climate change has been suggested often (Lorenzoni and Pidgeon 2006; Weber 2010; McDonald et al 2015; Reser et al 2014). This hypothesis is rooted in literature which shows that people can use direct personal experiences, in addition to secondary sources (e.g. scientists, media), to understand otherwise abstract risks. Such personal experiences may help anchor people’s understanding of climate change by making the risk more concrete and familiar (Smith and Joffe 2013; Bickerstaff and Walker 2001; Spence et al. 2012).

Many studies have now examined connections between variations in actual and perceived temperature and perceptions of climate change (e.g. Capstick and Pidgeon 2014; Zaval et al. 2014; Howe et al. 2013; Li et al. 2011). However, while climate change is expected to lead to aggregate global temperature increases, one of the principal ways it is likely to be made concrete for ordinary people at a regional level is through extreme weather events (EWEs) (Coumou and Rahmstorf 2012). This distinction is important because experiences with EWEs might hold the potential for more profound changes in people’s perceptions of climate change more generally. EWEs may act as a strong ‘signal’ or ‘focusing event’ (Renn 2011; November et al. 2009) whereby future climatic events are made more imaginable, indicating dramatic changes to familiar and local places, in turn heightening the sense of risk posed by climate change. EWEs are also often associated with changed socio-political contexts (media coverage, institutional responses etc.) which themselves constitute important influences on people’s perceptions (cf. Pidgeon et al. 2003).

The empirical evidence on this issue is mixed. In the first study to examine this question, conducted 3 years after major UK flooding in 2000, Whitmarsh (2008) reported no systematic relationship between respondents in flooded and non-flooded areas and their climate beliefs. More recent studies do however suggest that EWE experiences can increase belief in climate change and promote support for sustainable behaviour change (Myers et al. 2013; Spence et al. 2011; Broomwell et al. 2015; Taylor et al. 2014a; Lujala et al. 2015). However, a number of conceptual and methodological aspects of these previous studies deserve closer attention.

### Conceptual considerations

While there has been a focus on establishing empirical associations between EWE experiences and climate change perceptions, the mechanisms by which this might occur remain relatively untested. One aspect that has received some attention in the literature is the possible role of heuristics in understanding the process by which experiences might affect risk perceptions or engagement. Indeed, there are clear parallels between assumptions made about the role of EWEs impacting climate change perceptions and psychological literature on the availability heuristic, which suggests that people’s judgements about risk are influenced by the ease with which relevant events come to mind (Weber 2010; Keller et al. 2006; Taylor et al. 2014a). As such, experiences of an EWE might make climate risk more cognitively available or salient in people’s minds, particularly where such a link has also been made within the media or the statements of prominent figures. We already know that following flooding experiences, people are more likely to perceive future flood risk and to buy insurance (in effect, an adaptation to that experience) due to the increased salience of flooding (Browne and Hoyt 2000). Equally, if a person has experienced EWEs, this could lead to heightened salience of climate change, making it easier to envisage other ways in which climate change might affect them personally.

A second heuristic, potentially acting as a parallel mechanism, or mediator, of the relationship between experience and risk perceptions, is the affect heuristic (Finucane et al. 2000). Here it is proposed that experiential learning is facilitated through an emotional response, which heightens the importance of the experience and its subsequent influence on attitudes towards risk (Weber 2010; Keller et al. 2006), whereby it is easier to remember affect-laden events. The intense emotional responses known to accompany flooding experiences (e.g. Walker et al. 2011) provide a potentially powerful mechanism linking these events with responses to climate change, and in a way that is likely absent in other types of climate ‘experiences’ such as temperature changes. We would expect those with strong direct emotional experiences of extreme weather to be more likely to have heightened perceptions of the event and its possible related risks (in this case climate change).

The increased salience of climate change deriving from direct experience might also manifest in terms of other forms of engagement. For example, many studies on climate change perceptions have noted that climate change ‘issue salience’ is low when compared with other issues (e.g. the economy, migration, health; see Pidgeon 2012; Taylor et al. 2014b; Capstick et al. 2015b). Accordingly, direct experiences might influence the relative importance of climate change in relation to other priorities in life, alongside its personal salience as a risk issue. Previous research, by contrast, has tended to focus primarily on changes to epistemic beliefs (i.e. whether people accept climate change is a physical reality; see Myers et al. 2013). It would be fruitful, therefore, to take a broader view of personal engagement with climate change, representing a shift of focus – moving away from asking whether ‘seeing is believing’ and asking instead whether experiences lead to engaging and acting.

A final consideration in the current study is to examine not only personal salience and mitigation intentions, but also attitudes towards wider climate policies as well as adaptation intentions to ostensibly unrelated climate events (in particular heatwaves). It is conceivable that if an EWE experience heightens climate change issue salience among those affected, this might in turn translate into responses to other types of climate risks. Such a link between EWE experience and wider climate adaptation has received very limited attention to date (Reser et al. 2014; Blennow et al. 2012) but has potentially important implications for future resilience planning and wider public engagement (Moser 2014).

The discussion above leads to the following four hypotheses. People with direct personal experience of the 2013/14 winter flooding, when compared to a sample without direct experience, will have: **(Hypothesis 1 – Issue Salience)** increased issue salience of climate change in general, and increased salience relative to other issues; **(Hypothesis 2 – Risk Perceptions)** higher perceptions of general climate change risks; **(Hypothesis 3 – Emotional Engagement and Climate Change Mitigation)** higher levels of emotional engagement with the flooding events, which in turn will be positively associated with (as a mediating variable) higher climate change risk perceptions, mitigation intentions and policy support; and **(Hypothesis 4 – Non-Flood Adaptation Intentions)** higher intentions to engage in non-flood related climate adaptation measures (in this case regarding heatwaves).

### Methodological considerations

In order to outline our approach to testing these hypotheses, it is important to draw attention to several shortcomings in the methodology used within the research literature to date. First, an important limitation has been the wide-ranging way in which experience has been conceptualised, measured and interpreted. Many studies have used ambiguous or loosely-defined constructs or left participants to judge for themselves what might constitute ‘experience’ of extreme weather (e.g. Dai et al. 2015; Rudman et al. 2013; Spence et al. 2011; Taylor et al. 2014a; van der Linden 2014; Lujala et al. 2015; Whitmarsh 2008). Other studies have asked participants to state whether or not they have personally experienced global warming (e.g. Akerlof et al. 2013; Blennow et al. 2012; Broomwell et al. 2015; Myers et al. 2013; Reser et al. 2014), which in effect confounds both experience and a global warming belief in a single item – this is particularly problematic where an attempt is made to infer a causal link between these two constructs. Likewise, items that directly include an explicit ‘climate change attribution’ element (i.e. whether an event is attributed to climate change) can shed light on the extent to which people believe climate change has manifested in their own lives (or the extent to which events are consciously attributed to climate change), but subsequent linkages with perceptions or actions on climate change are more problematic to interpret. This is because, in part, such items are particularly susceptible to response biases: those already concerned about or accepting of climate change are more likely to consider particular EWEs to represent manifestations of it (Corner et al. 2012; Capstick and Pidgeon 2014). In other words, it is entirely possible that self-report of ‘experience’ simply acts as a substitute for people’s climate change beliefs more generally. It is not surprising under such circumstances to find that this type of ‘experience’ measure is sometimes the most significant predictor of other climate change belief measures.

More generally, we also know that information relevant to climate change tends to be interpreted according to pre-existing social, cultural and political beliefs (Corner et al. 2012; McCright and Dunlap 2011; Kahan 2014). Even if measures of experience do not explicitly refer to climate change, they are still susceptible to substantial biased reporting if they are left open to interpretation. This includes measures that do not specify the type of experience (e.g. property damage, travel disruption, emotional reaction), the type of EWE (e.g. flooding, drought, storms), or that include vague or long timescales. If undefined then the participant must themselves decide what constitutes a relevant EWE experience. In the first study to show an association between self-reported flood experience and climate change attitudes, Spence et al. (2011) concede that their interpretation suffers from precisely this causality issue. People already concerned about climate change may have been more likely to report that they had been impacted by ‘flooding in their area’; a suggestion which subsequent studies have corroborated (Blennow et al. 2012).

Taking account of these methodological concerns, we argue that clear and concrete measures of experience are necessary if research is to draw valid conclusions about how those experiences might affect beliefs (rather than vice versa). Although all self-report measures have limitations, it is likely that precisely-worded measures of direct physical experience and material impacts of an event, well-defined in terms of concrete personal effects and damage, are less susceptible to biased reporting, providing the best proxy available for direct measures of ‘objective’ experience^1^.

Additionally, a much-overlooked methodological consideration is questionnaire structure, particularly the potential influence that the order of question presentation might have on responses. If one is interested in the extent to which a particular experience affects attitudes towards climate change, then measures gauging this experience should be placed subsequent to these more subjective measures. This is because the placing of a measure early on in a questionnaire has the potential to confound findings through inadvertently leading respondents to consider this experience when reporting on their climate beliefs. As a result of this consideration, we placed key measures of climate beliefs as the very first items on the survey, so that these could be elicited independently from any mention of flooding^2^.

The present study seeks to examine the four hypotheses above, using a survey-based approach, while also addressing the methodological issues raised in this section. We apply a quasi-experimental design comparing a nationally representative British sample to individuals who had directly experienced the 2013/2014 UK flooding, taking care to: (a) measure key climate change perceptions using both prompted and unprompted questions *before* making any reference to extreme weather or flooding; and (b) employ a highly specific measure of direct flooding experience which is unlikely to be influenced by a person’s beliefs or preceding responses about climate change.

## Methods

The survey instrument was developed by the research team and refined after input from the social research company (Ipsos Mori) and advisory panel. The full questionnaire is detailed in Capstick et al. (2015a).

### Sampling

Computer Assisted Personal Interviews were conducted between 28 August and 31 October 2014 by experienced Ipsos Mori household interviewers and took 35 min on average to complete. The study design incorporated a nationally representative quota sample with 1002 interviews. Sample points were selected randomly using Double Output Areas (OAs). An OA represents the lowest level at which census information is published, and on which demographic quotas can be set. The Double OAs were stratified by social grade and rurality within regions. Quotas were set on age, gender and working status based on the local population of the Double OA, as published in the 2011 Census. Half of all interviews were completed on weekday evenings or at weekends. No incentives were offered.

To obtain an additional sample of respondents with direct experience of the flooding, five flood-affected areas were chosen to represent heavily affected areas with diverse experiences. These areas were chosen after consultation with the study’s advisory panel and Ipsos Mori, who had knowledge of where the flooding had occurred during the specified time frame. Media reports and data from the UK Environment Agency were then used to verify that flooding had indeed occurred in the sampled postcode areas (Environment Agency 2014)^3^. The five areas included the City of Hull, adjacent to the river Humber (*n* = 200), an area along the River Thames west of London between Sunbury and Windsor (*n* = 199), along the River Severn between Tewkesbury and Gloucester (*n* = 198), in the town and region of Aberystwyth in Ceredigion, Wales (*n* = 200), and along the coast at Dawlish in Devon (*n* = 198).

### Defining a flood-affected sample

The survey measured the occurrence of clearly defined, direct physical impacts of flooding on participants. Respondents were asked:

“Was your current or previous property affected by the floods between November 2013 and February 2014? This could include any land surrounding your home such as a garden or drive. If you live in a flat it might include communal areas such as a car park or hallway. Please also answer yes if you stopped the water from flooding your property by using some form of flood defence such as sand bags or a flood gate.”

Respondents who reported direct impacts on their property included 135 respondents from the flood-affected areas and 27 respondents from the national sample (*n* = 162)^4^. We compared this materially and directly affected sample to the national sample (*N* = 975: the nationally representative sample minus those who reported being directly affected).

We opted to compare these two samples because this provides a clear distinction between those with and without direct flood experience. The remaining participants in the oversampled flood-affected areas that are not included in this analysis are likely to have had close encounters with the flooding in other ways (e.g. through friends and family; Paranjothy 2011). These experiences are important but more susceptible to biased responding and are outside the scope of the current analyses.

The two samples naturally vary in terms of their geographic distribution across Great Britain (the national sample was drawn to be representative across regions, whereas the flood-affected sample was primarily drawn from the five specified flood-affected areas). We cannot fully rule out that the geographic location of the samples contributed to any perceptual differences found (see section 4), however by drawing the flood-affected sample from multiple locations, we reduce the likelihood of one location-specific factor affecting the analysis (e.g. the flood-affected areas included coastal and non-coastal areas). We further conducted an analysis comparing the two samples on a number of demographic and other variables. These comparisons reveal only small differences across the two samples, with the exception of social grade, an indicator of affluence (Online Resource 1 for the full analysis). This variable is therefore subsequently included as a covariate in all analyses.

### Measures

Items and response scales used to measure climate change perceptions are detailed in Table 1.

**Table 1:** Comparisons between the flood affected (*n* = 162) and national samples (*n* = 975) across climate change perception measures

Theorised construct	Item wording and response scale	Mean (+/- SDs)	Analysis of variance results
Concern	How concerned, if at all, are you about climate change, which is sometimes referred to as ‘global warning’? (very concerned, fairly concerned, not very concerned, not at all concerned)	National = 2.83 (0.83) Flood affected = 3.04 (0.81)	F(1,1119) = 6.71 *p* = 0.010
Temporal distance	When, if at all, do you think the UK will start feeling the effects of climate change? (We are already feeling the effects, in the next 20 years,25 years, 50 years, 100 years, beyond the next 100 years, never)	National = 5.92 (1.56) Flood affected = 6.18 (1.37)	F(1,1081) = 3.82p = 0.051
Threat to self, UK, others	People in developing countries: How serious a threat, if at all, is climate change to each of the following? (5-point scale: strongly agree to strongly disagree)	National = 3.77 (0.95) Flood affected = 3.84 (0.93)	F(1,1077) = 0.49p = 0.483
	The UK as a whole: How serious a threat, if at all, is climate change to each of the following? (5-point scale: strongly agree to strongly disagree)	National = 3.17 (0.94) Flood affected = 3.37 (0.93)	F(1,1109) = 5.30p = 0.021
	You and your family: How serious a threat, if at all, is climate change to each of the following? (5-point scale: strongly agree to strongly disagree)	National = 2.73 (0.97) Flood affected = 3.08 (1.07)	**F(1,1119) = 17.82,** ***p*** **= 0.000**
Threat to local area	My local area is more likely to be affected by climate change than most other places in Britain. (5-point scale: strongly agree to strongly disagree)	National = 2.32 (1.04) Flood affected = 3.40 (1.18)	**F(1,1114) = 133.40,** ***p*** **= 0.000**
Personal issue salience	Scale (Cronbach’s α = 0.794): - Discuss climate change with your family and friends? : How often, if at all, do you currently do each of the following? - Read and think about climate change? : How often, if at all, do you currently do each of the following? (5-point scale: strongly agree to strongly disagree)	National = 3.78 (1.41) Flood affected = 4.23 (1.31)	**F(1,1128) = 9.82p = 0.002**
Day-to-day worry	I worry about climate change on a day-to-day basis. (5-point scale: strongly agree to strongly disagree)	National = 2.29 (1.13) Flood affected = 2.44 (1.20)	F(1,1117) = 1.51p = 0.220

Test results in bold indicate significant differences between samples after Holm’s sequential Bonferroni adjustments to p-values (new significance level *p* < 0.006). Analysis of variance results are the follow-up tests to a significant multivariate analysis (F(8983) = 15.96, *p* < 0.001), and include social grade and age as covariates. All variables are coded whereby higher numbers respond to higher levels of concern or agreement

Behavioural intentions were measured using a four-item scale (Cronbach’s α = 0.66): “In the next few years, how likely or unlikely would you be to do each of the following? Change to a ‘green’ energy supplier which would reduce the impact on the environment from the electricity you use in your home; cut down the amount you travel by car; buy appliances that are more energy-efficient; reduce the amount of energy you use at home” (5-point scale: very likely to very unlikely).

Policy support for climate change mitigation actions was measured using a three-item scale (Cronbach’s α = 0.65): support for “road pricing schemes to reduce traffic in town and city centres; tax increases to pay for more renewable energy; the UK signing up to international agreements to limit carbon emissions” (5-point scale: strongly support to strongly oppose).

Non-flood related climate change adaptation intentions were measured using a two-item scale (Cronbach’s α = 0.83): “Find out about how to avoid health problems during heat waves; seek advice on how to cope with heatwaves and water shortages” (4-point scale: ‘It is very unlikely I would do this’ to ‘I am intending to do this’ with an additional option of ‘I don’t think this is relevant to me’).

A negative emotion response scale (Cronbach’s α = 0.83) combines answers to the questions “when you think about the floods how strongly, if at all, have you felt each of the following emotions?” (10 point scale: not at all to extremely) for five emotions (sadness, anxiety, anger, helplessness, distress).

## Findings

We compare the directly flood-affected sample (*n* = 162) to a nationally representative sample without flooding experience (*n* = 975) to determine the influence of flood experience on climate change issue salience and perceptual measures as discussed in section 3.1.

### Hypothesis 1: Issue salience

**Fig. 1:**
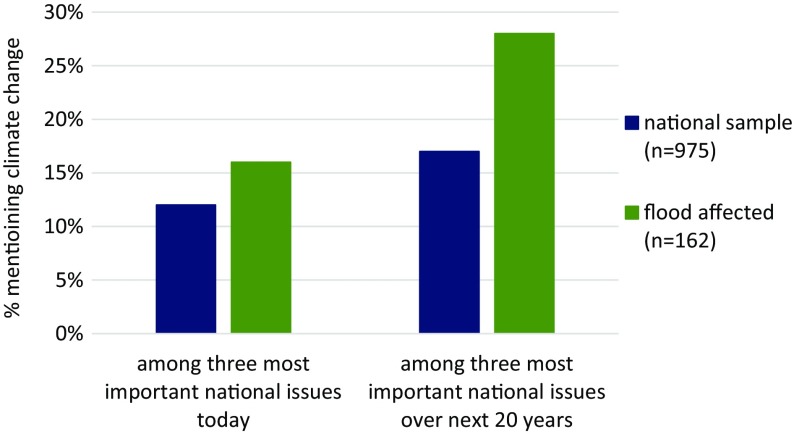
Proportion of respondents spontaneously offering climate change as one of the three most important issues today, and over the next 20 years

At the very beginning of the survey, and prior to any mention of climate change or flooding, we asked respondents to state the three most important issues facing the UK *today*, and *in the next 20 years.* Responses were coded blind to respondents’ self-reported experience of flooding. As these questions were open-ended (respondents answered in their own words) we consider these measures of unprompted issue salience (Hypothesis 1). Binary logistic regression analyses on responses to these questions reveal the following: the proportion of those presenting climate change as among the top three most important issues *today* did not differ significantly between the samples (b = 0.26, odds ratio = 1.30, *p* = 0.28). However, the odds of flood-affected respondents mentioning climate change within the top three issues *over the next 20* years was 70 % higher relative to the national sample (b = 0.53, odds ratio = 1.70, *p* < 0.01); see Fig. 1. This finding indicates that climate change was indeed a more salient topic for those who had directly experienced flooding, although only when judged at longer timeframes. Males and those in more affluent social grades were also more likely to spontaneously mention climate change as one of the most important issues facing Britain in both questions (see Online Resource 2 for details).

### Hypothesis 2: Risk perceptions

Hypothesis 2 predicts higher climate risk perceptions in the flood-affected sample. In order to compare concern and other relevant risk perception variables between the flood-affected and national samples, multivariate analysis of variance was used. Here we consider several standard constructs to take a broad view on engagement with climate change, including general concern about climate change, perceived temporal distance of climate change, and risk perceptions in the form of threat to developing countries, to the UK and to the self and family as well as to the local area. Personal engagement with climate change is measured using two indicators; one captures more cognitive aspects (the extent to which people read and talk about climate change) and one captures more affective aspects (worry about climate change day-to-day).

Differences between the two samples on these measures were all in the expected direction, and significant overall in a multivariate analysis incorporating all dependent variables (F(8983) = 15.96, *p* < 0.01); follow-up analyses of differences in individual items are displayed in Table 1 (Bonferroni correction applied).

We observe a pattern of differences whereby there was heightened personal salience of climate change within the flood-affected sample compared to the national sample (corresponding to indicators of personal issue salience, and threat to self and one’s local area). Differences in climate change concern are of marginal significance (*p* < 0.01 not meeting the adjusted significance threshold for multiple tests). However, the two samples did not differ on items of less immediate relevance at the personal level (threat to the UK and developing countries and perceived temporal distance). A lack of differences on some measures may be due to a ceiling effect; for example, most people across both samples believed that the UK is already experiencing climate change impacts. The only indicator of personal engagement with climate change that did not differ across the two samples was that of worry about climate change on a day-to-day basis.

In conclusion, our data shows those in the flood-affected sample displaying a heightened personal risk of climate change, however the samples do not differ significantly on whether it is an immediate risk causing worry on a daily basis.

### Hypothesis 3. Emotional engagement and climate change mitigation

Having identified these differences between the two samples, we next examine which key perceptual variables mediate the effect of experience on climate relevant mitigation intentions and policy support (Hypothesis 3). We especially consider the role played by *heightened personal salience* of climate change as found in the previous analysis. Accordingly, to capture affective processes more directly, we include a measure of *negative emotional responses* to the flooding itself. As expected, the flood-affected sample reported higher negative emotional responses (mean = 4.65, SD = 2.56) compared to the national sample (mean = 3.90, SD = 2.12), F(1,1128) = 88.58, *p* < 0.01).

To test the hypothesised relationships, mediation analysis was conducted. Specifically the analyses examine the direct and indirect effects of flood experience on mitigation intentions and policy support, with negative emotions associated with the flooding, climate change concern^5^ and personal issue salience included as mediators. We used the PROCESS add-on for SPSS (Hayes 2013) and allowed all mediator residuals to covary and estimated the direct effect of the independent variable on the dependent variable so that indirect effects were not overestimated. The method used bootstrapping to resample the data (5000 times) in estimating indirect effects. Variables were coded so that higher values indicated greater levels of that factor, for example greater concern or greater levels of personal engagement.

We examined *mitigation* responses using two outcome variables – behavioural intentions to act on climate change and support for climate change policies^6^.

**Fig. 2:**
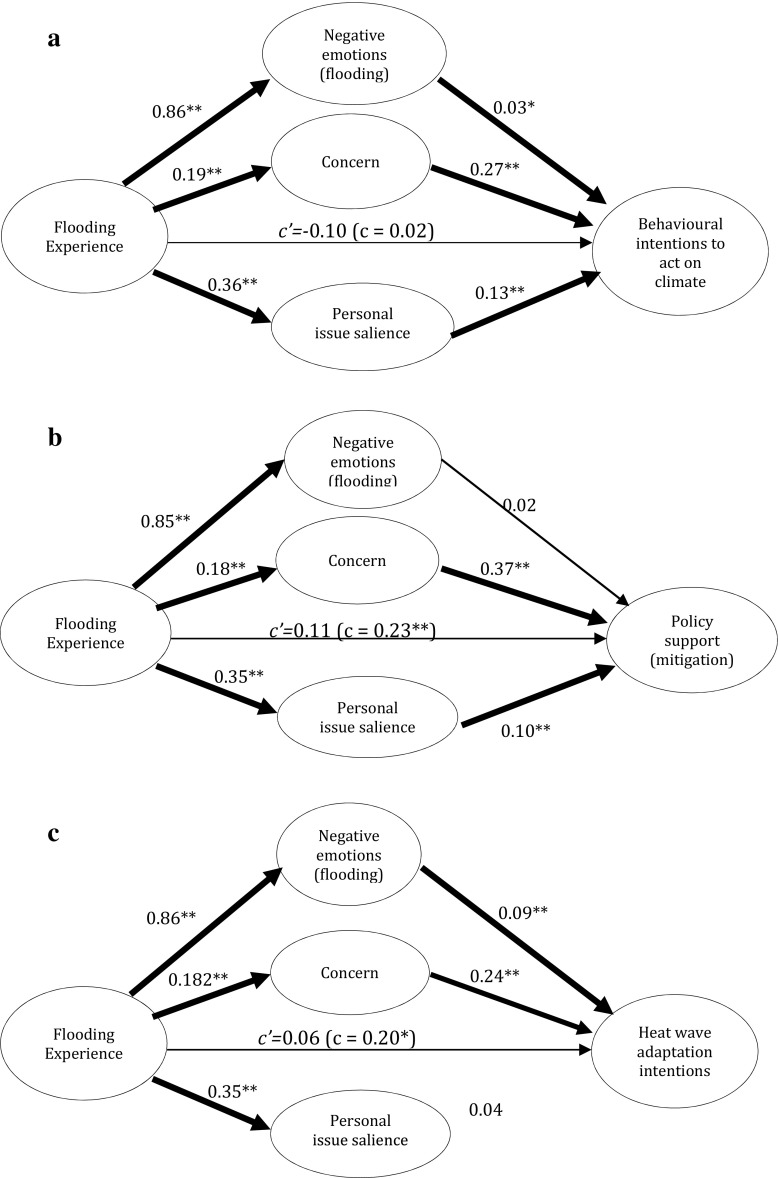
Mediation models examining flooding experience’s impact on (**a**) behavioural intentions to act on climate change and (**b**) support for climate change mitigation policies and (**c**) non-flood related adaptation intentions (heat wave). Values provided are unstandardized beta weights indicating the strength of the relationship between variables. *Heavy lines* indicate a significant pathway (* = *p* < 0.05, ** = *p* < 0.01); *c’* represents the direct effect of experience on behavioural intentions/policy support (holding other factors constant), *c* represents the total effect of experience on intentions/support

For the *behavioural intentions* measure the full model explained 25 % of variance (R^2^ = 0.25, F(7,1115) = 53.05, *p* < 0.01; 14 deleted cases due to missing data, *N* = 1123) and significant indirect effects were found for all three of the mediators (Fig. 2a). Direct flooding experience exerted an indirect effect on behavioural intentions through higher negative emotions associated with the EWE (B = 0.02, SE = 0.01, 95 % confidence intervals 0.006–0.046), increased concern (B = 0.05, SE = 0.02, 95 % confidence intervals 0.013–0.092), and higher personal issue salience with climate change (B = 0.05, SE = 0.02, 95 % confidence intervals 0.018–0.079). There was no direct effect of experience on behavioural intentions. Key demographic variables (gender, age and social grade) were included in the analysis to ensure any effects found were not due to their influence^7^.

For *policy support* the full model accounted for 22 % of variance (R^2^ = 0.22, F(7,1111) = 45.39, *p* < 0.01; 18 deleted cases due to missing data, *N* = 1119). Direct flooding experience exerted an indirect effect on policy support through increased concern (B = 0.07, SE = 0.03, 95 % confidence intervals 0.015–0.123), higher personal issue salience with climate change (B = 0.04, SE = 0.01, 95 % confidence intervals 0.013–0.068). No indirect effect was found for negative emotions associated with the EWE (B = 0.01, SE = 0.01, 95 % confidence intervals −0.006–0.039); see Fig. 2b.

Here there was a direct effect of experience on policy support, which became non-significant once the indirect effects are accounted for, indicating that the mediators included explain the relationship observed. Key demographic variables were included to ensure any effects found were not due to their influence^8^.

### Hypothesis 4: Non-flood adaptation intentions

We also examined attitudes towards *adaptation*, focussing on non-flood related adaptation measures to test whether the increased salience of climate change found in the earlier analysis translates to other domains, in this case intentions to *prepare for a heat wave* 9 *;* see Fig. 2c. The same mediation model as reported in section 3.3 was used with the dependent variable changed to heat wave adaptation intentions. The full model accounted for 10 % of the variance (R^2^ = 0.10, F(7,1109) = 16.64, *p* < 0.01; 20 deleted cases due to missing data, *N* = 1117). Direct experience of flooding exerted an indirect effect on greater intentions to prepare for a heat wave through negative emotions associated with the flooding (B = 0.08, SE = 0.03, 95 % confidence intervals 0.037–0.140) and heightened concern about climate change (B = 0.05, SE = 0.02, 95 % confidence intervals 0.011–0.088). No effects were found for personal issue salience (B = 0.02, SE = 0.01, 95 % confidence intervals −0.003–0.045).

We did find that flooding experience in this case directly (albeit marginally) influenced adaptation intentions; however this becomes non-significant once mediators are accounted for indicating the mediators explain this relationship. Key demographic variables were included to ensure any effects found are not due to their influence^10^.

## Discussion

We used a rigorous methodological design to establish whether the 2013/14 UK winter flooding influenced climate change perceptions, and to assess the processes by which this occurred. Our analysis revealed a heightened personal salience of climate change issues, an increased concern about climate change, and the experience of negative emotions following flooding experiences. These perceptual changes not only translate into an increased propensity to take personal climate change mitigation actions, but also appear to trigger broader intentions beyond this, including support for mitigation policies and intentions to adapt to another potential climate impact (heatwaves).

We conclude that climate change becomes more cognitively available following flooding experiences, in line with the operation of the availability heuristic (Weber 2010; Keller et al. 2006; Taylor et al. 2014a) – also indicated by the finding that those with flooding experience perceive higher levels of personal and local area threat from climate change. This mechanism is important both for prompting personal behavioural intentions to act on climate change and for the support of climate-related policy. Personal issue salience is not a significant mediator for the relationship between flooding and heatwave adaptation however, most likely due to differences in issue focus; here increased concern and emotional responses following flooding experiences appear to be more important drivers of intentions to act. Indeed, by including a measure of negative emotion associated with the flooding event, we provide insight into the role these might play in the link between EWE experience and subsequent climate change engagement. Here we find that negative emotions mediated flooding experiences for behavioural intentions to both mitigate and adapt to climate risks, but this relationship was not observed for policy support. This suggests that people’s support for climate change policies is less affect-driven compared to personal action intentions, perhaps given that these constitute more abstract decisions about wider society (see Ziegler and Tunney 2012).

### Implications

The influence of flooding experience on climate change perceptions in this study was found to be important for a range of theoretically predicted constructs, although it should also be noted not across all measures. Nonetheless, it is important to understand how people’s experiences with extreme weather might influence their engagement with climate risks, especially in a context where EWEs are predicted to increase in frequency and severity (Huntingford et al. 2014). The findings speak to the importance of a societal discourse or narrative around extreme weather that includes climate change. Communicating about the risks of flooding and other EWEs may provide a powerful overarching narrative about climate impacts for engaging local and wider publics about rising climate risks (Wallace 2012; Messling et al. 2015). While communicating about the need for policy responses, and the importance of personal action to mitigate and adapt to climate change must be carried out with sensitivity to the substantial harmful consequences encountered by those whose lives have been disrupted by these events, we suggest communications should not shy away from making links between extreme weather and climate change, where appropriate.

Of course, the exact process of how someone comes to link one or more extreme weather events to climate change is likely to vary by type of experience, over time, and by other aspects of the background social and political context. For example, in some cases this might not be a particularly conscious process, and may develop gradually over time. Equally, we would argue that this is less likely to occur if the media, elite figures, local groups or other societal actors are silent on the matter (Corner 2014). Accordingly, incorporating climate change into discussions about extreme weather early (i.e. in advance of events), coherently and alongside actionable advice on how to prepare is likely to be beneficial for the future resilience of at-risk communities (Messling et al. 2015).

In this regard, climate change communication is often framed solely as a matter of fostering mitigation action. But, in the face of rising climate risks, support for climate related policies and the nature of public adaptation responses will become increasingly important for social sciences research (Moser 2014; Dessai and Sims 2010; Pidgeon and Fischhoff 2011). We find for the first time that EWE experience in one domain translates to intentions to prepare for other types of climate impacts such as heat-waves. This remains a promising line of further inquiry, particularly since adaptation actions, unlike many mitigation options, are often located at the local or personal level. Theoretically, however, there remains much more work to be done to understand the precise psychological and social processes by which our perception of climate change becomes more relevant at a local and personal level (Brugger et al. 2015).

### Limitations and future considerations

Methodologically, this and previous studies demonstrate that the ‘experience–climate change perceptions’ link is a difficult relationship to untangle. In order to draw definitive casual conclusions longitudinal studies are needed. However, even for such studies it could be argued that the act of repeatedly taking part in ‘climate change surveys’ might itself affect people’s perceptions (e.g. raising issue salience, triggering motivated reasoning processes) in ways which would be absent in people who have not been part of such a cohort.

For practical reasons we were not able to utilise a longitudinal design in the current study, however we believe this study demonstrates improved methodological rigour by focusing on a specific EWE and clearly conceptualising and defining ‘experience’, a consideration which has been mostly absent in the previous literature. There are also a number of further lines of inquiry which might yield important further insights. These might include examining effects of different types of experiences (e.g. indirect experiences, such as involvement in a community response to an EWE) and different types of EWEs (drought, heat waves; e.g. Lefevre et al. 2015). A further extension of the current research would be to examine how political and personal ideology interacts with EWEs, as this has been shown to be a key determinant of climate change perceptions in both the UK and US (see e.g. Capstick et al. 2015b). Myers et al. (2013), for example, have suggested that experience might only influence people’s belief in climate change if they do not have strong sceptical beliefs to begin with.

Adding to the growing research in this area, the current study provides further confidence that direct experience of having been flooded has the potential to influence a person’s engagement with climate change. Finally, this study indicates that suitably framed narratives around climate impacts, extreme weather and rising climate risks might offer a productive route to greater engagement of various publics with climate change.

## Electronic supplementary material

Below is the link to the electronic supplementary material.

Online Resource 1 (DOCX 20 kb)

Online Resource 2 (DOCX 18 kb)

## Footnotes

1 Given that, for both ethical and practical reasons, direct observation of the physical impacts upon research participants during an event is highly unlikely.

2 In addition, participants were recruited to the survey without any mention being made of either climate change or flooding.

3 Postcode data of flood-affected areas as well as the database used to verify flooding had occurred is available from the authors upon request.

4 Postcode prefix data was used to verify if these 27 respondents were living in areas that had been affected by flooding in the specified time frame. Using Environment Agency data, 13 respondents were verified to be living in affected areas, 2 had postcode data missing and 12 could not be verified in this way. It is possible either that these 12 respondents nevertheless experienced flooding, or that they misreported their flood experience. Given the specific nature of the question used to measure experience, and acknowledging that the Environment Agency database of flood affected areas may not have comprehensively pinpointed all affected properties, we opted to still include these 12 respondents in our flood-affected sample.

5 We opted to include climate change concern as a mediator even though it was not found to be significantly different across the national and flood-affected sample in section 3.2. This decision was made because climate change concern has consistently been shown to be an important predictor of general risk perception in previous research in this area (Roser-Renouf and Nisbet 2008). In addition, the analysis in 3.2 applied strict Bonferroni adjustments to the significance threshold; climate change concern was at a marginal level of significance using this method.

6 Means and standard deviations for these variables are as follows: Behavioural intentions to mitigate climate change in the national sample (Mean = 3.64, SD = 0.78) and flood-affected sample (Mean = 3.71, SD = 0.83); support for climate change mitigation policies in the national sample (Mean = 3.33, SD = 0.94) and the flood affected sample (Mean = 3.61, SD = 0.88)

7 Gender did not significantly predict behavioural intentions (b = .-0.05, SE = 0.042, p = 0.22), however age (b = .-0.01, SE = 0.001, p < 0.05) and social grade (b = .-0.04, SE = 0.019, p < 0.05) negatively predict behavioural intentions whereby younger respondents and those in more affluent social grades are more likely to intent to change their behaviour.

8 Gender did not significantly predict policy support (b = 0.09, SE = 0.052, p = 0.07), however age (b = .-0.004, SE = 0.013, p < 0.05) and social grade (b = .-0.07, SE = 0.024, p < 0.05) negatively predict policy support whereby younger respondents and those in more affluent social grades are more likely to support climate policies.

9 Means and standard deviations for heat wave adaptation intentions in the national sample (Mean = 2.49, SD = 1.00) and flood-affected sample (Mean = 3.67, SD = 1.21).

10 Gender (b = 0.02, SE = 0.070, p = 0.760) and social grade (b = -0.04, SE = 0.032, p = 0.232) did not significantly predict adaptation intentions, however younger respondents displayed stronger intentions to adapt to heat waves (b = -0.004, SE = 0.02, p < 0.05).
