# Supplementary material for: Experience of extreme weather affects climate change mitigation and adaptation responses
Source: Clim Change. 2016 Oct 24;140(2):149–64. doi: 10.1007/s10584-016-1837-4 (PMC7175646; doi:10.1007/s10584-016-1837-4)
Supplement: Supplementary file 1 — (DOCX 20 kb) [file 10584_2016_1837_MOESM1_ESM.docx]

**Online Resource 1 for:**

**EXPERIENCE OF EXTREME WEATHER AFFECTS CLIMATE CHANGE MITIGATION AND ADAPTATION RESPONSES**

**Journal: Climatic Change**

Christina Demski^1^*, Stuart Capstick^1^, Nick Pidgeon^1^, Robert Gennaro Sposato^1^, Alexa Spence^2^

^1^Understanding Risk Research Group, School of Psychology, Cardiff University, Cardiff CF10 3AT, UK

^2^Horizon Digital Economy Research/School of Psychology, University of Nottingham, Nottingham NG7 2TU, UK,

*Corresponding author: [DemskiCC@cardiff.ac.uk](mailto:DemskiCC@cardiff.ac.uk), Tel. +44 (0)292087 6020, Fax +44 (0)292087 4679

Comparing the flood-affected sample (n=162) and the national sample (n=975)

The flood-affected sample has slightly more male respondents (61% males) proportionally than the national sample (52% males); χ^2^(1) = 5.06, p<0.05. The samples do not differ in terms of mean age, t(1130) = 0.75, p=0.45 or household tenure, χ^2^ (1) = 0.90, p=0.34. We also compared the samples in terms of social grade, a variable based on the occupation of the Main Earner according to the International Standard Classification of Occupations. Compared to the national sample, the flood-affected sample includes a significantly higher proportion of respondents categorised as social grades AB (more affluent social grades; 41% in the flood-affected sample and 29% in the national sample), and significantly less in C2 (less affluent social grade; 15% in the flood-affected sample and 21% in the national sample); χ^2^(3)=13.74, p<0.01.

We found no strong evidence for differences in voting intention between the national and flood-affected samples. Whilst a chi-square test comparing across all voting intentions (Conservative, Labour, Liberal Democrat, UKIP, other) is significant (Χ^2^(4)=17.651, p<0.01) this reflects differences only between Liberal Democrat voting intentions between the two samples, for which the overall number of respondents are low (n=31 in the national sample compared to n=14 in the flood-affected sample, representing about 6% and 15% of respondents respectively). Previous research in the UK suggests that Conservative voting intention is most closely associated with attitudes towards climate change (Whitmarsh, 2011). We found no differences between the two samples considered here; the flood-affected sample was comprised of 26% Conservative voters, as compared to 28% in the national sample.

To further rule out any differences across the samples prior to the study, we also compared the two samples on their reported environmental identity and cultural worldviews (controlling for age and social grade in both analyses). Environmental identity is measured using two statements “Being environmentally friendly is an important part of who I am” and “I think of myself as someone who is very concerned with environmental issues” which formed a reliable scale (Cronbach’s α = 0.82). Respondents indicated their agreement with each item on a 5-point agreement scale whereby higher numbers indicate agreement. Although the flood-affected sample reported slightly stronger environmental identity (M=3.82 SD=0.87) compared to the national sample (M=3.67 SD=0.92) this difference was not found to be significantly different, F(1,1121)=3.23, p=0.07.

Cultural worldview was measured using 6 items derived from Capstick and Pidgeon (2014) and drawing on earlier work by Rippl (2002) and Leiserowitz et al. (2010). Cultural worldviews reflect a person’s preferences for differing social structures (e.g. one based on equal sharing of resources versus individual freedom to accumulate wealth) and are known to predict perceptions of climate change (Xue et al., 2014). Principal components analysis of the six items led to a two-factor solution corresponding to individualism and egalitarianism; factor scores were used in subsequent analysis (cf. DiStefano et al., 2009). A multivariate analysis of variance revealed that the two samples were not significantly different on either the egalitarian or the individualism factor scores, F(2,1004)=0.70, p=0.50.

Additional references

DiStefano C, Zhu M, Mindrila D (2009) Understanding and using factor scores: considerations for the applied researcher. Pract. Assess. Res. Eval. 14:1-11.

Leiserowitz A, Maibach E, Roser-Renouf C, Smith N, Dawson E (2010) Climategate, Public Opinion and the Loss of Trust. New Haven, CT. http://environment. yale.edu/climate/publications/climategate-public-opinion-and-the-lossoftrust.

Rippl S (2002) Cultural theory and risk perception: a proposal for a better measurement. Journal of Risk Research 5(2):147–165.

Xue W, Hine DW, Loi NM, Thorsteinsson EB, Phillips Wj (2014) Cultural worldviews and environmental risk perceptions: A meta-analysis. Journal of Environmental Psychology 40: 249-258.
