# Supplementary material for: Experience of extreme weather affects climate change mitigation and adaptation responses
Source: Clim Change. 2016 Oct 24;140(2):149–64. doi: 10.1007/s10584-016-1837-4 (PMC7175646; doi:10.1007/s10584-016-1837-4)
Supplement: Supplementary file 2 — (DOCX 18 kb) [file 10584_2016_1837_MOESM2_ESM.docx]

**Online Resource 2 for:**

**EXPERIENCE OF EXTREME WEATHER AFFECTS CLIMATE CHANGE MITIGATION AND ADAPTATION RESPONSES**

**Journal: Climatic Change**

Christina Demski^1^*, Stuart Capstick^1^, Nick Pidgeon^1^, Robert Gennaro Sposato^1^, Alexa Spence^2^

^1^Understanding Risk Research Group, School of Psychology, Cardiff University, Cardiff CF10 3AT, UK

^2^Horizon Digital Economy Research/School of Psychology, University of Nottingham, Nottingham NG7 2TU, UK,

*Corresponding author: [DemskiCC@cardiff.ac.uk](mailto:DemskiCC@cardiff.ac.uk), Tel. +44 (0)292087 6020, Fax +44 (0)292087 4679

**Supplementary Table 1.** Binary logistic regression analysis of climate change issue importance *today* (Nagelkerke R^2^=0.03) and *in 20 years* (Nagelkerke R^2^=0.04).

|  | **Issue importance for *TODAY:***  Spontaneous mention of climate change  (0=no 1=yes) | | | |
| --- | --- | --- | --- | --- |
|  | B (SE) | Odds ratio | 95% confidence intervals | Significance level |
| Experience (national sample)   - flood affected sample | 0.26 (0.24) | 1.30 | 0.81-2.07 | p=0.28 |
| Gender (female)   - male | 0.50 (0.19) | 1.66 | 1.15-2.40 | p<0.01 |
| Social Grade | -0.21 (0.09) | 0.81 | 0.68-0.96 | p<0.05 |
| Age | 0.001 (0.01) | 1.00 | 1.00-1.01 | p=0.40 |
|  | **Issue importance *in 20 YEARS:***  Spontaneous mention of climate change  (0=no 1=yes) | | | |
|  | B (SE) | Odds ratio | 95% confidence intervals | Significance level |
| Experience (national sample)   - Flood affected sample | 0.53 (0.20) | 1.70 | 1.15-2.52 | p<0.01 |
| Gender (female)   - male | 0.43 (0.16) | 1.54 | 1.13-2.11 | p<0.01 |
| Social Grade | -0.23 (0.07) | 0.80 | 0.69-0.92 | p<0.01 |
| Age | 0.001 (0.004) | 1.00 | 0.99-1.01 | p=0.87 |
